# Vital Force

**Published:** 1897-06

**Authors:** J. M. Selfridge

**Affiliations:** Oakland, Cal.


### VITAL FORCE.

J. M. Selfridge, M. D., Oakland, Cal.

Previous to the time of Hahnemann the practice of medicine was a series of barbaric methods, much of which was prevalent at a time fresh in the memory of some of us who are now living. This was due to false theories, taught by Galen and others, and, as a consequence, purgation, salivation, blisters, the lancet, the seaton, the moxa, and the actual cautery were the sovereign remedies for almost every ill to which flesh is heir.

Instead of accepting the theory advocated by Hahnemann one hundred years ago, the allopaths have been laboring ever since, with a vain hope of discovering something peculiarly their own. But, as often as one theory was advanced it was replaced by another, until within the last two decades they have anchored themselves on the theory that germs are the cause of all diseases. But, alas! the practice which this theory suggested, when put to the test, so increased their death rate that their most courageous practitioners stood appalled at the unscientific results. The remedies used to kill the germs proved fatal to their patients, and, consequently, the germicidal treatment had to be abandoned. But, although their hopes were not realized, they still cling to the theory, notwithstanding the impracticability of the remedies it suggests.

Instead of the theory that germs are the cause of all diseases, we have the theory advanced by Hahnemann in *The Organon of the Art of Healing*, viz., that "Diseases are produced only by the disturbed vital force."

This theory was so different from the views held by the profession at the time it was uttered, and is so even now, with very few exceptions, that it was, and is to-day, challenged by the old school of medicine. There are also some of those who call themselves homeopaths who believe in a pathological basis for all diseases. But, with here and there an exception, all those who wear the badge of the homeopathicians accept it without question, and, in some things, go even farther than Hahnemann himself.

The fact that the theory advanced by Hahnemann is so different from any other that has preceded and followed it, makes it desirable that the search-light of modern science should be thrown upon it to determine, if we can, whether this radical declaration of principles will bear such an examination. With this thought in view, it will be well to examine some of the statements made by Hahnemann that bear on this subject, and then compare them with the facts of science as taught at the present time.

Hahnemann, at the outset, recognized the fact that man is a dual being, having a material organism so entirely dominated by what he calls a spiritual or spirit-like body, that the former can only become diseased by morbidly disturbing the latter.

This thought is first announced in the introduction to *The Organon*, on page twenty, where he says: "It was almost impossible for the common school of practitioners, in contemplating and judging of a disease, or in seeking the indications for its cure, to free their minds from these ideas of materiality, or to recognize the nature of an organism both spiritual and material, as a being so highly potentiated that the modifications of its life, called disease, manifested by sensations and functions, must alone be regarded as conditioned and affected by means of dynamic (spirit-like) influences, and that such sensations and functions could be affected by no other cause." And again on page twenty-three he says: "But the essential nature of diseases and their cure cannot accommodate themselves to such dreams, or to the convenience of physicians; diseases will not cease to be (spirit-like) dynamic aberrations of our spirit-like life, manifested by sensations and actions—that is, they will not cease, for the sake of those foolish and groundless hypotheses, to be immaterial modifications of our sensorial condition (health)."

It is a question in my mind whether Hahnemann, when he uses the terms spiritual and spirit-like, really means what is generally understood at the present time by the words spirit and spiritual. For it is difficult to understand how any person can believe that an immaterial essence, which is characterized by the absence of the properties that distinctly belong to matter, can become diseased.

From statements made in the introduction to *The Organon*, as well as in the body of the work, it is evident that he did not regard vital force as an independent spiritual essence like the human soul or spirit, for he speaks of it as "spirit-like"—that is, so minute in substance and so unseen in its operations as to be like or similar to spirit. From what follows in these pages it will be seen that he regarded it much in the same sense as physicists of the present day do force in general, as an inherent property of matter which acts automatically like cohesive, capillary, and gravitative attraction.

The first time Hahnemann uses the phrase "vital force" is in the introduction to *The Organon*, page twenty-eight, where he says: "Nay, this unreasonable vital force rashly receives into the body these chronic miasms (psora, syphilis, sycosis), the greatest tormentors of our earthly existence." On page thirty-four he says: "The vital force is capable of acting only in harmony with the physical arrangement of our organism, and without reason, insight or reflection, was not given to us that we should regard it as the best guide in the cure of disease." And on page thirty-five he calls it the "instinctive, unconscious, and unreasoning, but automatic vital force." In Section 10 of *The Organon* he says: "The material organism, without vital force, is incapable of feeling, activity, or self-preservation. This immaterial being

(vital force) alone, animating the organism in a state of sickness and health, imparts the faculty of feeling, and controls the functions of life."

There are those who hold the opinion that vital force and life are identical. But, taking this section, coupled with his statements in the introduction, it seems that Hahnemann did not so regard it; but merely as an agent that controls the functions which are set in motion by life. Dr. Boericke says that "The vital force is the intermediate agent between the spirit and the body, enabling the spirit to dwell for a time in its material bodily clothing." That "it is not the very seat of life, but only the connecting medium between the rational spirit, the true living man, and the outer material covering by which man takes cognizance of this material world."

L. S. Beale, in discussing this subject in his book *How to Work with the Microscope*, says: "The facts of the case teach us that a peculiar agency or force compels matter to assume temporarily the peculiar state characteristic of bioplasm or living matter, but of living matter alone. I venture to call this vital power. It is just as difficult to form a positive conception of this wonderful power as it is of gravitation or electricity."

It seems to me that this section gives a pretty good description of the functions of the nervous system, and suggests the thought that vital force and nerve force are nearly identical.

In Sections 11 and 12 Hahnemann lays down the doctrine, in unmistakable terms, that "diseases are produced only by the morbidly disturbed vital force."

That Hahnemann did not regard vital force as a spirit in the sense in which it is used at the present time, but rather as a substance so highly potentized as to be spirit-like, is evident, and for this reason. He describes it in precisely the same language he uses in speaking of his potentized remedies, which he stoutly maintains are material. In Section 16 he says: "Our vital force, that spirit-like dynamis, cannot be reached nor affected except by spirit-like (dynamic) process, resulting from the hurtful influences of hostile agencies from the outer world acting upon the healthy organism, and disturbing the harmonious process of life. Neither can the physicians free the vital force from any of these morbid disturbances—*i. e.*, diseases, except likewise by spirit-like (dynamic, virtual) alterative powers of appropriate remedies acting upon our spirit-like vital force; perceiving this remedial power through omnipresent susceptibility of the nerves of the organism."

It will be observed that "the hurtful influences of hostile agencies from the outer world" that disturb the vital force, and the remedies that cure, are material, yet he describes them in the same words he uses when speaking of vital force.

Wherever Hahnemann speaks of anything that is exceedingly minute he characterizes it as "spirit-like." For example, in discussing mental diseases, he says in Section 216: "In short, the disorders of the coarser bodily organs are transferred, as it were, to the almost spiritual organs of the mind." And in Section 288 he does not leave us in doubt as to what he means by the words "spirit-like." He says: "The effect of medicines in liquid form penetrates and spreads through all parts of the organism with such inconceivable rapidity, from the point of contact with the sensitive nerves supplying the tissues, that this effect may, with propriety, be defined as spirit-like (dynamic or virtual)."

In Section 20 Hahnemann teaches that a "spirit-like power" is "concealed in drugs;" and in Section 128 he tells us that by means of "trituration and succussion," "the powers hidden and dormant, as it were, in the crude drug, are developed and called into activity in an incredible degree."

There are those in the homeopathic school who claim that the curative force of drugs, during the process of potentization, is separated from the drug material, and becomes an independent, immaterial force, which, by means of "proper manipulation," can be transferred to inert substances, as alcohol, water, and sugar of milk. Now, although Hahnemann used these vehicles in making his potencies, he did not teach that the curative principle of drugs became an independent, immaterial force—*i. e.*, entirely freed from the original drug material. What he taught was just the reverse of this, and he was right, for the ideas he advocated seventy or more years ago on the potency question are in perfect harmony with the teachings of modern science, namely, that matter is infinitely divisible.

Modern science enables us to demonstrate more clearly why the potentization of drugs increases their curative powers. It teaches that by the divisibility of matter, its molecules, not only, but the ultimate atoms are set free. And Dolbear suggests that by proper means the atoms may be divided, thus making matter absolutely divisible to infinity.

That Hahnemann did not regard his potentized remedies as immaterial forces is evident, for if we read Section 280, and especially Note 143, we will find that he distinctly states that his highest potencies, however minutely divided, are composed of drug material. "Let these ordinary practitioners," he says, "ask mathematicians to demonstrate the truth, that, although a substance be divided into ever so many parts, some portion of this substance, however minute, must still constitute each one of these parts; that the most inconceivable minute fractional particle never ceases to be something of the original substance, and hence, that it never can become nothing." Let me emphasize this by repeating the statement that nothing can be clearer than that Hahnemann regarded the highest attenuations of homeopathic medicines as material, yet so finely comminuted as to be beyond the powers of human observation, and, therefore, as he so often expresses it, "spirit-like."

It is evident that he regarded all forces as potencies of some material substance, for we read in Note 143 that "physical sciences will teach them that there are great forces (potencies) which are imponderable, like heat, light, etc.,* and that, consequently, these must be far lighter than the medicinal contents of the smallest homeopathic doses."

While Hahnemann, doubtless, knew what he meant by the terms vital force, dynamis, dynamic, spirit-like dynamis, and spirit-like (dynamic) process, he uses them so frequently and so interchangeably as to be somewhat confusing. It will be of interest, therefore, to pass them in review and determine, if we can, their scientific meaning at the present time.

By the word vital is meant of or pertaining to organic life. By organic we mean "having or consisting of organs." It, therefore, applies to animals or plants.

Life—what is it? It has been defined as "that condition in which animals and plants exist, with capability of exercising their natural functions." It is also called the "vital principle or animal soul."

By force is meant that which can produce, change, or destroy motion. But, as to the essential nature of force, we know absolutely nothing, because we can neither see nor grasp it. "We simply know there must be a cause for certain effects produced." These effects are motion and rest, if there be such a condition as absolute rest.

By vital force, then, we mean a kind of "agency that compels matter to assume the characteristics of bioplasm," and it is present in both animals and plants.

Force is inseparable from matter; at least so far as we are able to determine; and it is, without doubt, an inherent property of matter, as much so as the attraction of gravitation or cohesion, which, in fact, are forms of force, and are

*At the time Hahnemann wrote Note 143, " heat, light, etc.," were held to be material substances.

common to all bodies of matter, and are eternal and unchangeable.

The statement that force is inseparable from matter will be called in question by those who hold the opinion that the curative force of drugs can be transferred, "by proper manipulation," to inert substances; and the familiar example of the ability to transfer magnetic influence from the natural magnet to soft iron and steel may be quoted. That force can be transferred, under suitable conditions, from one body of matter to another, is not denied; but this example does not prove that drug force can be transferred to substances which are medicinally inert; for it is evident that magnetism is permanently transferrable only to substances that are molecularly like the magnet. It is true that soft iron may become magnetized while in contact with a magnet, but it parts with its magnetism as instantaneously as it receives it, for the reason that its molecules are destitute of what is called in science "coercive force," and for that reason it cannot become a permanent magnet. But steel, which possesses a large amount of "coercive force," and is molecularly like the magnet, not only receives but retains magnetism. It is, therefore, always selected for permanent magnets. The original magnet, however, although it imparts magnetism to other bodies of matter, seems to lose none of its magnetic force. It is evident, therefore, that to transfer force permanently from one substance to another, they must be molecularly similar.

It is a scientific fact that "the identity of elementary matter resides in the atom, while the identity of compound matter resides in the molecule." Hence, if it were possible to separate force from the material substance of a drug with which it is connected, the drug would lose its identity.

For example, if it were possible to divide and subdivide Arsenicum to such a degree that the ultimate atoms would be reached, and, then, if we could apply what has been called

"proper manipulation," by fluxion or otherwise, until its peculiar or specific force was transferred to some inert vehicle, where would be our Arsenicum? Of course, it would have lost its identity, and, therefore, would not be Arsenicum. A drug, to be the vehicle of a particular energy, must retain the atomic and molecular structure peculiar to that drug, or it might, by chance, be something else. What makes one drug differ from another drug? There are seventy or more different elementary substances, and the molecules of each drug are composed of from three to one thousand atoms of one or more of these elements, variously combined, and in nearly every instance these combinations are secured only by chemical action, as, for example, "carbon, which in its pure crystallized form we know as a diamond, appears as petroleum when combined with hydrogen, as carbon dioxide gas when combined with oxygen, and as sugar when combined with both hydrogen and oxygen;" and, yet, the carbon loses neither quantity nor identity. It is evident, therefore, that drugs differ from one another because of the various combinations of the atoms of one or more of these elements.

Force plays an important part in all the operations and combinations of nature; but it cannot be maintained that force *per se* is the drug, for we know absolutely nothing of force except as it manifests itself through these molecular combinations. That the effect of a drug like Silicea, for example, can be increased by any process that will set its molecules free, is a fact known to every intelligent homeopathic physician; but to claim that force can exist or manifest itself in a certain manner, as, for example, like Silicea or Arsenicum, without the material atoms and molecules of these drugs, is to make an assertion without proof. There is no scientific foundation upon which to base such an assumption.

The doctrine that drug force can be separated from its material substance was an unnecessary invention, for the reason that there is such an infinite number of molecules, to say nothing of the ultimate atoms in every form of matter. For example, careful computations have demonstrated that there are 78,690,000,000,000,000,000 of molecules in a quantity of matter no larger than the head of a pin. Now, if it were possible to place this immense number of molecules, there would be enough to plant 12,128,565,484 of them in every quarter of an inch of ground in forty acres of land, square measure. If, in addition to this, we could separate these molecules into their ultimate atoms, there would be sufficient material present to saturate the highest potencies known to the imagination of the most superlative enthusiast.

Force is in constant action. It is either pushing or pulling, and it acts automatically; consequently there is no such condition as absolute rest in the universe. This is true of all matter, whether animate or inanimate. Every molecule in the great blocks of granite that give form to our mountains, and every molecule in the ribs of steel that make the frame-work of those massive "liners" that plow the Atlantic and Pacific Oceans, are in a state of quivering motion in their inconceivably small spaces, so small that it is asserted by physicists that if a person could count ten millions of molecules every second of time it would take him 250,000 years to count the number of molecules in a speck of matter the size of a pin's head; and, yet, each one is moving backward and forward, bounding and rebounding from its neighbors, who, like itself, are in ceaseless motion—a motion so rapid as to be beyond conception.

From considerations like these it is evident that motion is as much of an inherent property of matter as is capillary, cohesive, or chemical attraction. Still, every thing that moves suggests the idea of force. That force which causes the molecules of rock and iron to move to and fro in their narrow beds, although an inherent property of matter, may be defined as inanimate force, or simply force, while that which is observed in matter of a higher order, that is, in organic matter, we call it vital force. Can it be denied that the latter is any less an inherent property of organic matter than that the former is an inherent property of inorganic matter? I think not. Each was implanted in its respective sphere by the Great Architect of Nature, when both the organic and inorganic forms of matter were created. It is evident, therefore, that force manifests itself in two ways: as a power that is inherent in dead or inert matter, like earth, rocks, metals, etc., and in a higher degree, as vital force, which manifests itself in all forms of organic matter, including plants and animals.

That branch of science which treats of force and the motions it produces is called dynamics.

Dynamics, then, means power—related to, or endowed with power. Dynamis is synonymous with power or force. Dynamics investigates the powers whereby bodies are put in motion, and the laws that govern them. In biology it relates to the vital forces—to the organism in action. Hence we have vital dynamics which relates to the action of remedial agents on the human organism not ascribable to either mechanical or chemical causes.

Vital force has been defined as a specific force, which is assumed to account for organic life and its phenomena. In evolution it is a form of energy conjectured to give rise to and control the phenomena of organic life. In biology, especially evolutionary biology, it is any force that aids in producing the phenomena of organic life.

Infidel biologists, like Haeckel and some others, teach that both force and life are evolved from matter.

All that scientists can really prove in regard to force and life resolves itself into pure conjecture. They approximate, but they know absolutely nothing about the nature or essence of either the one or the other. Essential knowledge belongs alone to the mind of the Infinite.

Those of us, however, who recognize the existence of a Supreme Being believe that when He constructed this vast universe He endowed matter with both force and organic life. By organic life I do not mean the spiritual or immortal part of man which cannot be corrupted by material contact, but I mean that kind of life which is common to plants and the lower order of animals, and also occupies the protoplasm in the primal cell of all vertebrates, including man. Were it not for a higher order of protoplasmic life, each primal cell would undoubtedly remain like an ameba—a distinct and independent cell, and there would be neither cell aggregation nor a scientific arrangement of the various organs. But, in the higher order of beings, there is a different kind of protoplasm, more complex and refined, and its offices are correspondingly of a higher order. This complex molecular structure is found only in the cells of the cerebro-spinal and ganglionic nerve centres; and without the force derived from the molecular action of these centres, there would be neither digestion, circulation, secretion, assimilation, nor growth in the animal body. This force is the great supervisor of all vital dynamics, and is very similar to, if not the identical vital force of Hahnemann.

It is undoubtedly true that this force is generated by the inherent vibrations of the molecules of the highly vitalized cells of these great centres; for, if their action be arrested by severe "shock," or other means, all the various organs cease their activity, and if the equilibrium of their molecules be not promptly restored, death itself may be the result. Vital force, like all force, is so dependent, and so interwoven with matter, that there can be no manifestation of its presence without matter. Neither can matter exist without force, for "there is no point in the universe where matter would cease to possess energy." And, so far as we know, the converse of this is equally true, force cannot exist without matter.

The interdependence of matter and force is well illustrated by a familiar example. Take the egg of the common barnyard fowl. In it we have the molecules of albumen, fibrin, etc., which, although in constant motion, resemble the ameba in this, they are not able to develop into anything different from themselves until a protoplasmic germ of a higher order—call it what you may, vital force or life force—is planted among them. And, even then, this germ is not only powerless to change the condition of these molecules, but they will in a short time undergo decomposition, unless they are warmed into increased activity by the vibratory motions of the molecules of matter exterior to themselves.

The same facts are observed in connection with a grain of corn or wheat. The germ, with its contained vital force, is present, but absolutely powerless to act until its molecules are made more active by the vibratory motions of the molecules of the sun or some other body of matter.

It is evident, therefore, that vital force is entirely dependent upon protoplasmic conditions for its ability to manifest its presence. In other words, unless the material conditions are normal we have an abnormal exhibition of vital force, and the result is a pathological condition.

Take the brain of a newly-born child. It is perfectly healthy, but there is no ability to manifest thought, or what is called mind. Why is this so? It is not that there is no vital force or brain cells present, but it is because the protoplasm in those cells is not fully developed. Not only so, but there is no connection between those cells, and, therefore, no concert of action, and the result is that no mind is secreted. Now, if we follow this newly-born child up to perfect adult life, we will find a condition of mind to correspond to the condition of brain development. And, if we continue our observations to extreme old age, we will find that as those brain cells become imperfect in their protoplasmic structure, we will observe a correspondingly weakened condition of mind, thus accounting in a scientific manner for the old adage, "Once a man, twice a child."

I know it may be claimed that the want of mental manifestation in the newly-born child is because the vital force has not had time to develop its brain substance. Now, granting that this is true, how do we explain the decadence of the senile mind? No one can deny that vital force is present, and, if present, why does not the mind continue in full vigor to the end of its earthly existence?

When the systolic action of the heart plugs one of the arteries of a hitherto perfectly healthy brain with an embolic clot, thus depriving a portion of it of its nutrient supply, why is there a failure of mental manifestations? Is it because there is no vital force present? Certainly not. It is because the brain cells are deprived of their accustomed nutriment, and, consequently, they are incapable of secreting thought or receiving impressions, notwithstanding the fact that the vital force is present to urge them to duty.

On the other hand, we may have a perfect development of brain cells and protoplasmic contents, and yet there will be no mental manifestations, unless the vital force be present and in active operation.

The conclusion, therefore, is obvious—vital force and organic matter are dependent the one upon the other, and there can be no normal activities in the organism unless both are present and in harmonious action.

From the examination we have given this obscure but very interesting subject we have seen that force is an inherent property of inorganic matter, and that vital force is also an inherent property of organic matter; and that vital force and nerve force, if not identical, are not only similar in their nature, but near neighbors in the organism; and, also, that motion plays a very important part in the functions of all organized bodies, and in inorganic matter also.

Now, since there is such an interdependence between motion and the molecules of matter, and since it has been shown that molecular motion is not dependent upon vital force, and since it has been shown that vital force is dependent upon matter for its ability to manifest itself to our senses, and since all growth has its initial point in the primal cell (which is what its contained molecules make it), and since all healthy as well as unhealthy conditions of an organized body are the result of the normal or abnormal movements of its molecules, we cannot avoid the conclusion that when disease manifests itself in an organized body it is due to the disturbance of its atomic and molecular motions. On the other hand, if the molecules of matter upon which the vital force depends for its ability to manifest itself to our senses be disturbed, it is evident that the vital force, with which they are so intimately connected, must also be disturbed. Therefore, the conclusion is inevitable—that, notwithstanding the fact that Hahnemann did not have the discoveries of modern science to assist him in his researches, the conclusions he reached were not far from the truth. Science not only confirms his statements, but demonstrates that the interpretation given to some of his teachings is erroneous. In this connection it will be of interest to notice some of the conclusions of science which are closely related to the thoughts expressed in these pages, viz., the manner in which heat is produced. As early as 1812, Sir Humphrey Davy announced that "The immediate cause of heat is motion." The modern statement is "that heat is kinetic energy; not evidently of the mass, since the hot body may be at rest, but of the molecules." That "temperature has to do simply with the speed of the molecular motion existing in a body." That "when heat is transferred to a body its temperature is raised—*i. e.*, the speed, and therefore the energy, of its molecular motion is increased. Conversely, as heat is withdrawn from a body, its temperature falls, the speed of its molecular energy decreases, and its kinetic energy is diminished." "The amount of heat necessary to raise the temperature of a body is proportional (1) to the number of molecules it contains, and (2) to the increase of the kinetic energy of a single molecule." In other words, heat in a body is due to the motion of its molecules.

From this it is clear that science controverts the theory of physiologists in regard to the manner in which animal heat is produced. It is well known that their theory is that animal heat is maintained by the oxidation of nitrogen, carbon, and hydrogen, and that of these elements the heat value of hydrogen is by far the greatest, and that this oxidation takes place chiefly in the substance of the various tissues, and that it is connected with the general process of nutrition and disassimilation, but this oxidation, it is claimed, "is not necessarily a process identical with combustion out of the body."

Pathologists hold the same theory, and teach that when abnormal heat is present in any part of the body of a patient it means combustion.

I have quoted these different statements for two reasons: (1) Because the molecular theory is in perfect accord with scientific thought, and fully explains the method of producing both normal and abnormal animal heat in the body of a patient; and (2) for the reason that the allopaths have adopted a system of practice based on the theory of combustion, which I believe to be as dangerous as it is unscientific.

The practice to which I allude is inspired by their terror of a high temperature, and their belief that it must be reduced at all hazards, without reference to whether the fever be inflammatory or typhoid.

The means they employ are chiefly the ice-bag, the cold bath, or powerful antipyretics, which are too frequently followed by heart failure and death.

The scientific method of treating a high temperature, whether inflammatory or otherwise, is to restore the molecular activity of a diseased organ to its normal condition. This is done, as will be shown further on, by administering a remedy whose action is similar, and in that way obliterate the disease upon which the high temperature depends "in its entire extent, in the shortest, most reliable, and safest manner."

The manner in which the most similar remedy relieves the organism of abnormal disturbance must be in touch with modern science, and be susceptible of a scientific explanation.

Science has demonstrated that all bodies of matter have "physical fields," which are capable of compelling other substances which are constructed molecularly like themselves to assume a similar state or motion as the body that produced the field. Now, since all medicinal substances must, therefore, have fields peculiar to themselves, and since, for that reason, they are capable of compelling other substances which are molecularly similar to assume like motions or conditions, it follows that a healthy field can only be established in a diseased organism by the action of a similar substance or medicine, the vibrations of whose molecules are similar to the vibrations of the molecules of the diseased organism.

To speak more specifically, this is accomplished through the ability of the atoms and molecules of the drug to penetrate the walls of the primal cells (where all activities commence in the organism), and by means of the medicinal force (which cannot be separated from the drug material) so to influence the vital force that the movements of the molecules which constitute the protoplasm of the primal cells are controlled, and their abnormal activities changed to normal movements, and thus the diseased organism is restored to perfect health. By some it is claimed that remedies act by elective affinity; but the trend of scientific thought at the present time seems to indicate that drug force has the power to change the polarity of the molecules of a diseased organism, and in that way restore them to their normal condition.

To state it more briefly: To cure disease, it is only necessary to restore the disturbed molecules of a diseased organism to their normal vibrations. To do this in a scientific manner it is necessary to administer an attenuated remedy in accordance with the law of similars, which is, without doubt, a law of Nature.

No greater truism was ever uttered than that by Dunham, when he said, "Homeopathy is the science of therapeutics."

